# Appraisal of the *Deqi* Concept among Contemporary Chinese Acupuncturists

**DOI:** 10.1155/2013/538476

**Published:** 2013-10-02

**Authors:** Sheng Chen, Shengnan Guo, Federico Marmori, Yanping Wang, Qi Zhao, Baokai Wang, Eunhae Ha, Yanhuan Miao, Li Xiang, Mingwen Zhao, Yuwei Huo, Yinan Nan, Li-an Liu, Jiping Zhao

**Affiliations:** ^1^Dongzhimen Hospital Affiliated to Beijing University of Chinese Medicine, China; ^2^European Foundation of TCM, Spain; ^3^Acupuncture Department, Medimar International Hospital, Spain; ^4^School of Acupuncture, Moxibustion and Tuina, Beijing University of Chinese Medicine, China; ^5^School of Preclinical Medicine, Beijing University of Chinese Medicine, China

## Abstract

*Deqi*, an important component of the traditional theory of acupuncture and moxibustion, is the key factor in determining clinical therapeutic effect of acupuncture. In this paper, based on the digging up, arrangement, and in-depth analysis of the famous contemporary Chinese acupuncturists' perspectives of *deqi*, the authors summarize the concept and manifestation, as well as the properties of *deqi*, and correlativity of *deqi* with acupuncture manipulation through reviewing modern clinical research. Proposals for more scientific and standardized acupuncture research are introduced to reexamine and restore the implication of *deqi* in combination with the clinical practice.

## 1. Introduction

Western medicine influence has played an important and challenging role in the development of modern acupuncture leading to the establishment of the integrative medicine school of thought. Modern acupuncturists in China have enhanced acupuncture theory, basing their work on scientific knowledge. Modern China has been the starting point of studies on the relationship between meridians and nerves. In spite of the tendency to integrate scientific rationale in the acupuncture theory, most doctors still acknowledge the importance of preserving the classical theory with its cultural background and extended classical bibliography. Therefore, modern acupuncturists in china, with their experience and viewpoint, embody the actual acupuncture and the link between past and present.

Chinese medicine has evolved over thousands of years, building up through the accumulation of clinical experiences. Experience and oral transmission are ineluctable pillars of Chinese medicine and acupuncture heritage. Since time immemorial to the present day, *deqi* has always been a key point in practice and research of the acupuncturist. The importance given to *deqi* derives from its clinical significance, as well as the practitioner's traditional and conservative views in feudal society that associate the difficulty of *deqi* technique with mystical beliefs that go beyond rational explanation. This paper is as a complete summary as possible of the experience of renowned Chinese acupuncturists since 1949 and their viewpoint about *deqi*. Our intention is to provide new elements for modern research and guidance for clinical application.

## 2. Materials and Methods

### 2.1. Object of the Study

The famous contemporary acupuncturists involved in our research are selected from the following:

1. those with honor given by the Ministry of Personnel of China, the Ministry of Health, and the Drug Administration of China, specializing in acupuncture;
2. acupuncturist expert experience-albums which are in great influence include albums such as the *Clinical Essentials of the contemporary Chinese Acupuncture* [1], *Clinical Guideline of Acupuncture and Moxibustion* [2], *Integration of contemporary Zhe Jiang Acupuncture Study *[3], *Collection of the Beijing Famous Acupuncturists* [4], and the *Essence of the Famous Acupuncturists *[5];
3. chief editors and subeditors of acupuncture and moxibustion textbooks.

A total of 140 acupuncturists were selected.

### 2.2. Source of the Literature and Search Strategy

The theory and the experience of the modern famous specialists were kept in 2 ways: one in a form of a network information database and one kept in a form of the literature of cultural relics. Our research combined these 2 sources in the method shown as follows:

1. the following electronic databases were searched, regardless of publication status: the Chinese National Knowledge Infrastructure Database (CNKI) (1949–2013), the Chinese Science and Technology Periodical Database (VIP) (1989–2013), the Chinese Biomedical Database (CBM) (1978–2013), the Wanfang Database (1985–2013), and PubMed Database (1966–2013). All searches ended in April 2013. The search terms included “the names of acupuncturists above,” “*de_qi*” (getting *qi*), “*qi_zhi*” (arrival of *qi or qi arrival*), and “*zhen_gan*” (acupuncture sensation or needling sensation);
2. collecting the literature of acupuncture and moxibustion with the names of acupuncturists above in the title.

### 2.3. Inclusion Criteria

Studies meeting the following three criteria were included: (1) taking “*de_qi*” (getting *qi*), “*qi_zhi*” (arrival of *qi or qi arrival*), or “*zhen_gan*” (acupuncture sensation or needling sensation) as subject; (2) concerning direct expression of personal experience or viewpoint; (3) quoting acupuncturists' consensus or professional opinion.

### 2.4. Exclusion Criteria

The following studies were excluded: (1) duplication: the same content with the same authors published in different journals; (2) mentioning “*de_qi*” (getting *qi*), “*qi_zhi*” (arrival of *qi or qi arrival*), or “*zhen_gan*” (acupuncture sensation or needling sensation), but without a critical point of view or without a comment.

## 3. Results and Discussion

### 3.1. The Literature Research and Study Selection

**Figure 1:**
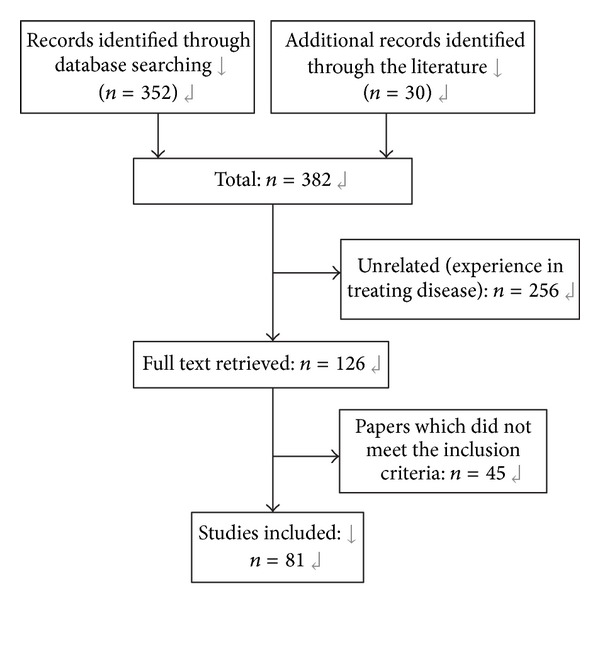
Flowchart of the literature search and study selection.

Our initial searches identified 352 references (344 from Chinese databases and 8 from English database) and 30 works of literature. After study selection, a total of 81 references (19 works of literature and 62 studies) were included (Figure 1).

### 3.2. Concept and Manifestation of *Deqi*

#### 3.2.1. Historical Origin of *Deqi*

The theory of *deqi* (getting *qi*) and arrival of *qi* originated from *The Yellow Emperor's Inner Canon*, which occurred many times in different chapters. It was elucidated more deeply in the following books such as *The Classic of Difficult Issues, The Great Compendium of Acupuncture and Moxibustion, The Ode of the Golden Needle, and Song to Elucidate Mysteries*. For two thousand years and through the various dynasties, the *deqi* concept has been central to academic thinking.

The purpose of acupuncture, moxibustion, or other forms of stimulation is the dredging of meridians and regulating of blood and *qi*. Even though *qi* does not have a material form, thus cannot be palpated, special importance is given to the regulating of its flow. Because scientific development and theories are being based on physical material, it is thus challenging for people not acquainted with eastern culture to understand such a concept [6]. The arrival of *qi* and *deqi *mentioned in *The Yellow Emperor's Inner Canon *stresses on the feeling of doctors but did not consider patient's sensation as *deqi* and *qi* arrival. Mention of patient soreness, numbness, pain, or similar sensations secondary to needle stimulation only appears in the literature at the end of the *Qing* Dynasty, in *The Inner Chapters of Acupuncture and Moxibustion* [7] which is to become the rudiment of *deqi* theory in actual clinical acupuncture today.

#### 3.2.2. Knowledge of *Deqi* from the Ancient Time to the Present Day

Dr. Cheng [8] considers the arrival of *qi* and *deqi* the same. The concept of *qi* contains the description of both doctors and patients feelings. This view is accepted by most acupuncturists nowadays, such as the description in Chinese acupuncture textbooks: *deqi, *which is called the “arrival of *qi*” in ancient time and is called the “needling sensation” nowadays. *Deqi* means when the needle has been inserted to the desired depth, and manipulation techniques such as lifting and thrusting or twirling and rotating are applied to obtain meridians sensation in the puncturing location” [9]. Needling sensation refers to patient's feeling of soreness, numbness, distension, heaviness, pain, formication, and electrical sensation around the acupoint when the needle is inserted. At the same time, the operator may feel tenseness around the needle” [10]. Dr. Li [11] believes that *deqi* is a feeling and a reaction between the relevant feelings of patient and doctor, which cannot be considered as *deqi* when it lacks in either one.

The view of *deqi* in the ancient medical literature differs from the current acupuncture and moxibustion circle. The ancients described it as the tenseness feeling beneath the operator fingers, while the current acupuncture and moxibustion circle pays more attention to the feeling of patients, which often includes soreness, numbness, distension, and heaviness. Modern researchers [12] divided the patients' feeling into thirteen types such as pain, soreness, deep oppression, heaviness, distension, chirobrachialgia, numbness, stabbing pain, dull pain, and the feelings of warmth, cold, spasm, and others. Simultaneously, they observe the occurrence of the frequency and intensity of needling sensation shown as follows: sore sensation, oppression sensation, tingling, numbness, and dull pain are more common, while warm sensation and cold sensation are less. Other scholars [13] made a clinical investigation and found that distension, soreness, electrical sensation, and numbness sensation have separately accounted for 94%, 81%, 81%, and 78% of the most common needling sensations, respectively. It is necessary to point out that needling sensation is a new term mentioned by the modern scholars who combined Western medicine knowledge with acupuncture research. It is beneficial for us to intuitively recognize all kinds of acupuncture stimuli. However, in the actual clinical, all kinds of needling sensation had difficulty in guiding the reinforcing and reducing methods. Thus, many physicians put forward their own views on the relationship between the needling sensation and *deqi*.

#### 3.2.3. Indications of *Deqi* in Different Perspectives

(1) *Besides the Sensation of Soreness, Numbness, Distension, and Heaviness, Other Feelings Can Be Combined.* Doctors like Qiu [14], Wang [15], Cheng [16], Shi [17], Zhang [18], Ge [19], Wei [20], and so forth all consider that the needling sensation does not only include soreness, numbness, distension, and heaviness in a local area, but also sensation transmission along the meridians or the arrival of *qi* at pathological sites. Dr. Liu [21] has pointed out that the slow transmission of sensation such as soreness, numbness, distension, and heaviness differs from the rapid radiating feeling of numbness by stimulating the nerve. Dr. Sun and Dr. Gao [22] believe that if the doctor feels a sunken or tense sensation beneath the needle, it is an indication of *deqi*. Patient in this case must have a feeling of soreness and distention beneath the needling point, or even an outward spreading feeling. “It can also be observed visually,” says Wei [23], if the skin around the needle appears tense, with a raised or sunken phenomenon, which is also regarded as a form of *deqi*.

(2)* Emphasizing Deqi Lies in the Doctor's Feeling.* Dr. Peng [24] believes that some of the patients who do not respond sensitively to the needle insertion or the feeling of sunken or tension beneath the needle are the indication of *deqi*. Dr. Wang [25] considers that the indications of *qi* arrival do not only consist of the patient's and the doctor's feeling beneath the needle, but also include the feeling of the doctor's other hand when pressing the skin near the acupoint during acupuncture procedure. Dr. Feng [26] thinks that the present *deqi* beneath the needle is known by asking the patient, and there is a certain subjective conjecture for the patient's description of the needling sensation. If the *qi* beneath the needle is mainly recognized by the doctor's feeling, it is more concrete and easier to control the needling. Dr. Chen [27] considers that in some special cases such as coma, emptiness beneath the needle, or noncooperation of the patient that causes the patient to be unable to reflect subjective feelings, the doctor must carefully observe the objective indications such as the sinking and tension beneath the needle and moving up and down of the muscle or limbs. But the most important at this point, which should be taken as the primary evidence, is the efficacy of the treatment. Dr. Zhang [28] thinks that the *deqi* sensation beneath the needle, as well as the changes and recovery of the pulse after needling, is more important than the patient's sensation. The sensation of *deqi* beneath the needle of the doctor's hand and the patient's needling sometimes is synchronous but sometimes not.

(3) *Other Views.* Dr. Jin [29] considers that the sensations of soreness, numbness, heaviness, and distension are just some superficial feelings in the local area which cannot be equally indicated as *deqi*. Dr. Lai [30] thinks that, nowadays, habitually equalizing *deqi* to needling sensation, or seeing the strength of needling sensation as *deqi* and determining the efficacy, is a cognitive mistake. The general feelings of the patient such as soreness, numbness, distension, and heaviness are original, primary, and initiative. Only doctors who identify the pathogenesis can do the manipulation of the reinforcing or reducing methods to achieve the real sense of therapeutic effect of *deqi*. In addition, the clinical practice has shown that some patients can also get a good efficacy with weak needling sensation or even with no needling sensation at all. In the modern acupuncture, such as wrist-ankle acupuncture, intradermal acupuncture, and abdominal acupuncture, needling sensation is not required for the patients, but many diseases can be cured. Therefore, the concept of latent needling sensation is raised [31], in which, during the acupuncture treatment procedure, the patient does not feel any of the needling sensations such as soreness, numbness, distension, and heaviness, but instead the doctor has a feeling beneath his hand. There is an obvious change of the electric conduction amount that can be detected by the meridian detector, which is proved by responding to the tissue at the acupoint. And the clinical efficacy is considered to be the criterion of the judging of *deqi*.

Thus, it is obvious that the needling sensation is not the only manifestation of *deqi*. As we all know, many physiological functions related to meridian phenomenon cannot be directly perceived, such as blood circulation, nerve conduction, muscle discharge, electrical impedance of skin, and hormone secretion. Therefore, it is very normal that the functional activity of the meridian is not directly perceived by people [32]. Another study [33] has shown that the sensitivity of the needling sensation may be related to the individual differences of the secretion levels of endogenous opioid peptides and antiopioid. Dr. Liu and others [34] use a self-developed apparatus to conduct a quantitative analysis of the frequency, speed, time, intensity, and subtle changes of the acupuncture operator to indicate the *deqi* of acupuncture, which has been all the time reacted to the subjective concept, which can be objectively detected through the mechanical monitoring.

### 3.3. The Properties of *Deqi*

#### 3.3.1. The Identification of Upright and Pathogenic Properties of *Deqi*

As early as 2,500 years ago, it was recorded in *The Yellow Emperor's Inner Canon* that the arrival of grain's *qi *is referred to as a sensation of “comes slowly and softly” after *deqi* which has the feeling of relaxation and alleviation; conversely, the arrival of *xie qi *is referred to as a sensation of fierce after *deqi*, with an unsmooth and dull feeling beneath the needle or even unable to manipulate the needle, which is hard for the patient to tolerate. Dr. Cheng [8] explains the upright *qi* and *xie qi* as follows: “If some neuron gets sick due to the overexcitement, the reflectivity will be stronger with a second stimulation. Hence, the sunken and tense feelings beneath the needle are produced and cause *xie qi *which is a morbid state. If the disease-free nerve is punctured, the reflectivity is brisk and ease, which is the state of mildness, called the upright *qi*.” In combination with the clinical practice, Dr. Qiu [14] also explains that *xie qi* refers to acute pain such as stomachache, colicky pain due to gallstone or nephrolith, high fever, or spastic limbs, which the body condition is in an extreme tense and lead to a fierce response after needling and cause the sensation of tense and dull pain due to spasm and contraction beneath the needle. “The upright *qi* refers to when symptoms were relieved with acupuncture needle insertion and the needling sensation turns soft and keeps constant, neither of tension nor emptiness.” Dr. Tian [35] summarizes briefly that the upright *qi* is mild, while the *xie qi* is quick and tense.

#### 3.3.2. The Relationship between Deficiency, Excess, Cold, and Heat Syndrome and the Indications of *Deqi*

Dr. Wang [15] thinks that the patient with cold syndrome has mostly a dull sensation beneath the needle and feels sore, while the patient with heat syndrome has a sensation of tense and knotting beneath the needle and feels distensile; the patient with deficiency syndrome has a loose and slippery sensation and feels numb; the patient with excess syndrome has a resisting and knotting sensation beneath the needle with a feeling of spicy pain. Dr. Peng [24] raises that, in the elderly with deficiency syndrome, the best needling sensation is the change of emptiness to sinking and tension sensation with a heat sensation beneath the needle as the best result, while, for the strong patient with excess syndrome, the best needling sensation is the change of sinking and tension to emptiness sensation with a cold sensation beneath the needle. The needling sensation of soreness and numbness is normally obtained with the neutral manipulation.

#### 3.3.3. Feelings of *Deqi* in Different Parts

Dr. Yang [36] summarizes the feelings of *deqi* in different levels of the tissue during acupuncture as follows: when the needle is punctured into the dermal part, the pain is sensitive; when punctured into the vessels, a little pain is felt; when punctured into the fascia, slight distension is felt; when punctured into the muscles, soreness and distension are felt; when punctured into the nerve, numbness and radiating sensations are felt; when punctured into the periosteum, pain is felt. The summary of Dr. Li [11] is similar to the above opinion. Dr. Yu [37] points out that pain is the sensation which is easily aroused when the hand, foot, head, or face is punctured, and it is also one of the needling sensations. Dr. Zhang [18] thinks that there are different needling sensations when acupoints from different parts are punctured. For the acupoints on the limbs, chest, and abdomen, or back transport points, the needling sensations are mostly soreness, distension, heaviness, numbness, and so forth. After the 500 times observations by puncturing EX-HN 3 (*yìntáng*) and GV 20 (*bǎihuì*), Dr. Liu and Ji [21] summarize that when the superficial fascia layer is punctured, there are only a slight distension sensation and sunken and tense feelings beneath the hand. According to clinical experiences, Dr. Wei [23] mentions that when blood vessels are punctured, there will be a heat or a burning sensation. Dr. Huang [38] stresses on *deqi* in the superficial layer. When a special needling manipulation is done in the dermal layer, the patient will generally have a slight sensation of numbness, distension, or radiation. He thinks that the appearing of the sensation is also good for getting the sensation of *deqi* in the deep layer, so that the effect of dredging and unblocking meridians could be achieved in real sense.

These views are derived from clinical experiences by acupuncturists from several decades, and nowadays, they are gradually explained by modern researches [39–43]. Related studies show that acupuncture effect signals are mainly initiated by somatosensory receptors and afferent fiber in the dermal layer, hypodermis, muscle, aponeuroses, tendon, interosseous membranes, and the periosteum. Different types of nerve fiber relatively conduct different types of sensation; for example, soreness, dull pain, and hotness are transmitted by slow-conductive fibers, A*δ* and C fibers, numbness, and tingling by faster-conducting A*β*/*γ* fibers, and pressure is transmitted through multiple different types of nerve fibers. Morphophysiology has shown that nerve innervations and tissue structures are actually closely interrelated to each other. For example, A*δ* and C fibers are mostly distributed in tendons [44]. Therefore, pain sensation in the deep region may include soreness, heaviness, diffusion, and duration. Another research [45] has indicated that the acupoints are densely distributed in regions such as the top region of the head, temporal region, and the central part of the trunk and extremities. These densely distributed regions have outstanding fundamental substances required to stimulate the transmission. Therefore, mastering the characteristics of the needling sensation in different parts of our body tissues may help doctors to accurately locate the depth and angle of the needle and to stimulate diverse receptors in different tissue layers to impulse different types of conducting fibers for certain effect. It has a certain guiding significance in manipulation of acupuncture.

### 3.4. *Deqi* and Acupuncture Manipulation

#### 3.4.1. Relationship between *Deqi* and Traditional Reinforcing and Reducing Methods

Modern Chinese acupuncturists think that reinforcing and reducing methods can only be proceeded after *deqi*. Dr. Jin [29] has pointed out that, by classifying the characteristics of *deqi*, we can either decide to use the reinforcing or reducing method. In times of *qi* arrives slowly, the sensation beneath the needle should gradually be filled, which also means that grain's *qi* has arrived, so the reinforcing method should be carried out. If there is a compact and fast sensation during the arrival of *qi*, which means that *xie qi* has arrived, so the reducing method should be used. Dr. Zhang [46] also believes that the reinforcing or reducing method should be based on examining and evaluating the condition of patients somatic function. Dr. Lu [47] has pointed out that *deqi* is even reflected after the reinforcing and reducing methods. If the reinforcing and reducing methods have reached their own standard, reinforcing method requires the sensation beneath the needle to be tense and full, which was loose and puff before the procedure. The reducing method requires the unsmooth and tight sensation beneath the needle to be changed.

#### 3.4.2. Relationship between *Deqi* and the Quantity of Stimulus

Currently in China, many Chinese acupuncture specialists gradually show evidence of westernizing in the knowledge of the acupuncture theory [48]. Take Dr. Zhu [49] for example; she has proposed that the main theory of acupuncture and moxibustion treatment of a disease is by stimulating and adjusting the internal organ nervous system, especially by adjusting and controlling the function of the senior central nervous system. Dr. Zhang [28] has also mentioned that, from the modern medicine point of view, the function of acupuncture and moxibustion can be classified as “function of physics” (change in the morphological area) and “function of chemistry” (changes in the physiology, pathology, and biology areas). The therapeutic effect of acupuncture and moxibustion is likely to be carried out by regulating the neurohumor.

Dr. Yang [50] considers that the therapeutic effect of acupuncture is mainly carried out by moderate stimulation of physical effect. Dr. Huang [38] considers that, in most situations, the stimulation intensity is at direct ration relationship with the *deqi* sensation.

Dr. Cheng [51] considers that there is no difference in the reinforcing and reducing methods, but only in the stimulation intensity which can be adjusted by changing the strength of *deqi* sensation. Dr. Lu [47] considers that the theory mentioned prior does not entirely correspond in reality. This is because light stimulation can excite and strong stimulation can restrain nerves in terms of the nerve response to the stimulation, but we consider reinforcing and reducing methods in terms of meridians, *qi* and blood. Since the two theories exist in different bases, the two cannot be compared in the same platform. At the moment, there is not enough evidence to prove that nerves are equal to the meridians, so there still needs to be a further discussion on replacing reinforcing and reducing methods with the stimulation intensity. Dr. Jin [29] agrees with the above mentioned point of view and thinks that even though there is no common standard of acupuncture technique between ancient and modern times, we can cross-reference and apply it in clinical practices. Even though Dr. Yang [50] thinks that the quantity of needling stimulus decides the therapeutic effect, he repeatedly emphasizes that the relationship between reinforcing, reducing, and the intensity of the stimulation is not simple. These two concepts exist in intersecting and embracing relationships.

#### 3.4.3. Relationship between *Deqi* and Retaining Needle Time

In the times of *The Yellow Emperor's Inner Canon*, the concept was to take the needle out immediately once patients feel *qi *arrival, which brings about the end of the acupuncture treatment procedure. Doctors, who have inherited the original decree of *The Yellow Emperor's Inner Canon *including Dr. He [52], Dr. Cheng [53], and Dr. Peng [24], consider that there is a need to wait for *qi* arrival if you do not get the sensation after needling. Once after *deqi*, the doctor takes the needle out immediately, and there is no limit in the treatment time. Nowadays, there are only a small amount of doctors who retain the needle just the way as it is recorded in *The Yellow Emperor's Inner Canon* and today, the occupation standard for retaining the needle has become 20 to 30 minutes. Dr. Je [54] thinks that, in order to get the most ideal therapeutic effect, there is a need to retain the needle after *deqi* in order to keep *qi*, and maintain the needling sensation, and the quantity of the stimulus. Dr. Luo [55] considers that, in the treatment of some chronic pain illnesses, the needle should be retained for about 1 hour or even longer, to keep patients with a certain needling sensation and enough quantity of stimulus, so as to obtain a better effect. Dr. Guo [56] mentions that retaining needle time should be determined by the state and duration of illness.

#### 3.4.4. Relationship between *Deqi*, Acupuncture Therapeutic Effect, and Prognostic Prediction

Dr. Peng [24] considers that there is a most ideal needling sensation suitable for patients with different body constitutions and ages. Dr. Qiu [57] has also mentioned that different therapeutic effects can be gained just by changing the direction of the needling sensation in one acupoint. For example, RN12 *(Zh*ō*ng Wǎn) *acupoint which is selected in the treatment of stomachache requires the needling sensation to scatter around the surrounding region to relieve pain; needling sensation for treatment of vomit is required to transfer downward.

Dr. Yu [37] thinks that different classifications of needling sensation establish different therapeutic effect. Needling sensation such as numbness and electrical sensation is suitable in the treatment of excess syndromes and acute diseases; tic sensation is suitable for visceral ptosis and paralysis; Dr. Guan [58] has proposed “highly efficient needling sensation” which is a sensation with special therapeutic effect in treating certain diseases, such as in treatment of sciatica and needling sensation of GB30 *(Huán Tiào)* spread down to the foot which belongs to one of the highly efficient needling sensation.

Dr. Cheng [59] considers that the length of time required to *deqi* does not only influence the therapeutic effect but can also be used as a determination of patient's condition, treatment, and prognosis. “Faster the *deqi*, higher the rate of *deqi*, brings better the therapeutic effect”.

## 4. Questions and Expectations

*Deqi* is a specialized term used in acupuncture, and it has a significant role in selecting the needle manipulating methods, determining the therapeutic effect of acupuncture and body response. In the present, objectively and quantitatively standardizing the measurement for *deqi* state of the patient presents a significant challenge in this field [60].

The preliminary summary of famous contemporary acupuncturists' viewpoint about *deqi* can help the acupuncture practitioner, in combination with clinical practice, to revert to the original intention of *deqi* in future research. It is of vital importance to reexamine and restore the implication of *deqi* under more scientific and standardized acupuncture research for further guidance in exploring the therapeutic mechanism of acupuncture.
